# Hinged Plakin Domains Provide Specialized Degrees of Articulation in Envoplakin, Periplakin and Desmoplakin

**DOI:** 10.1371/journal.pone.0069767

**Published:** 2013-07-29

**Authors:** Caezar Al-Jassar, Pau Bernadό, Martyn Chidgey, Michael Overduin

**Affiliations:** 1 School of Cancer Sciences, University of Birmingham, Birmingham, United Kingdom; 2 Centre de Biochimie Structurale, CNRS UMR-5048, INSERM U-1054, Université de Montpellier I et II, Montpellier, France; Consejo Superior de Investigaciones Cientificas, Spain

## Abstract

Envoplakin, periplakin and desmoplakin are cytoskeletal proteins that provide structural integrity within the skin and heart by resisting shear forces. Here we reveal the nature of unique hinges within their plakin domains that provides divergent degrees of flexibility between rigid long and short arms composed of spectrin repeats. The range of mobility of the two arms about the hinge is revealed by applying the ensemble optimization method to small-angle X-ray scattering data. Envoplakin and periplakin adopt ‘L’ shaped conformations exhibiting a ‘helicopter propeller’-like mobility about the hinge. By contrast desmoplakin exhibits essentially unrestricted mobility by ‘jack-knifing’ about the hinge. Thus the diversity of molecular jointing that can occur about plakin hinges includes ‘L’ shaped bends, ‘U’ turns and fully extended ‘I’ orientations between rigid blocks of spectrin repeats. This establishes specialised hinges in plakin domains as a key source of flexibility that may allow sweeping of cellular spaces during assembly of cellular structures and could impart adaptability, so preventing irreversible damage to desmosomes and the cell cytoskeleton upon exposure to mechanical stress.

## Introduction

The plakin family of proteins connect the intermediate filaments that form the cell cytoskeleton to cadherin-mediated cell-cell junctions, and must be able to withstand mechanical stresses to provide integrity to tissues [1], [2]. Seven plakin proteins are found in mammalian cells, with envoplakin, periplakin and desmoplakin being associated with desmosomes in various solid tissues. Desmoplakin is found in all desmosomes, which are particularly abundant in cardiomyocytes and epithelial cells [3]. Its N-terminus interacts with other desmosomal proteins at the membrane and its C-terminus links to cytoskeletal proteins; severance of this link results in loss of cell-cell adhesion and severely compromises tissue integrity [4]. Envoplakin and periplakin initiate formation of the cornified envelope, a mechanical scaffold that replaces the plasma membrane of cells in the cornified outer layers of the epidermis and maintains water impermeability and resiliency of the skin [5], [6]. They associate with desmosomes and engage keratin intermediate filament proteins within differentiating keratinocytes [7], [8], [9]. Periplakin is also involved in cellular movement and attachment, and is downregulated in esophageal cancer [10]. However, their respective structural roles have yet to be differentiated.

Dysfunctional plakin proteins contribute to diverse diseases, with autoantibodies and mutations perturbing their activities with profound consequences. Envoplakin and periplakin are target antigens in the autoimmune blistering disease paraneoplastic pemphigus, and offer epitopes in their plakin domains [11]. Desmoplakin mutations cause the skin disorder disease striate palmoplantar keratoderma [12], [13] and arrhythmogenic right ventricular cardiomyopathy (ARVC), with the plakin domain being a hotspot with multiple identified pathogenic mutations [14]. ARVC is one of the most common cardiomyopathies and a cause of right ventricular arrhythmias, cardiac failure and sudden death in young adults, particularly athletes [15]. Inheritance of ARVC is usually autosomal dominant with variable penetrance. Autosomal recessive mutations in the desmoplakin gene cause Carvajal syndrome, a cardiocutaneous disorder that is characterised by palmoplantar keratoderma, woolly hair and heart disease [16], and lethal acantholytic epidermolysis bullosa, a skin blistering disease that causes catastrophic skin and fluid loss and early death [17]. Over 850 mutations in plakin family genes and interacting partners have been linked to diseases [14], underscoring the need to define their structural, dynamical and functional properties to guide diagnostic and treatment options.

**Figure 1:**
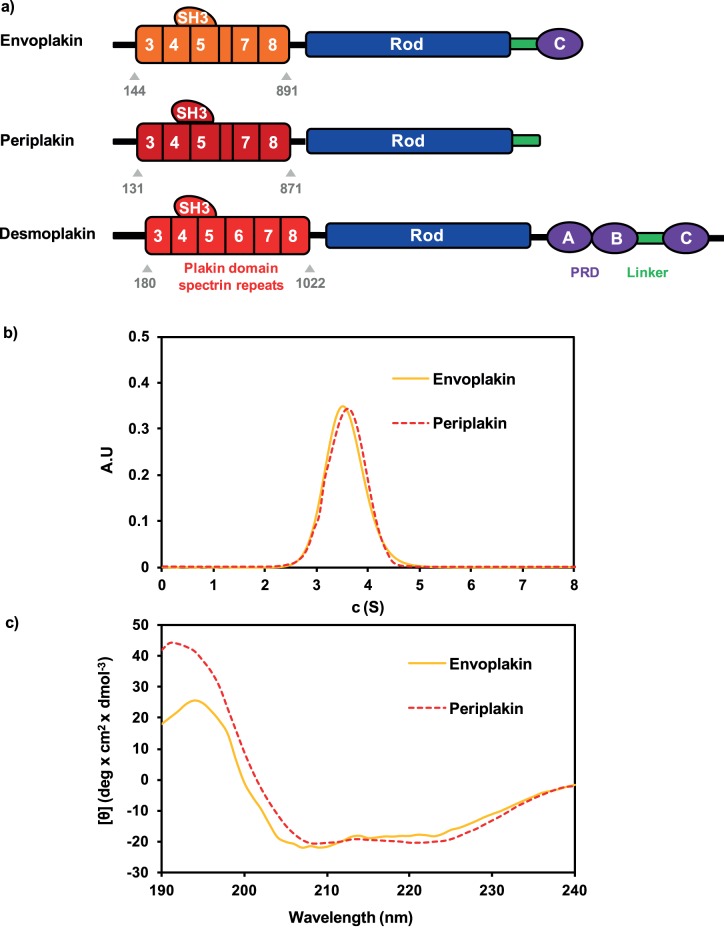
Structural and monomeric states of the plakin domains of envoplakin, periplakin and desmoplakin. (a) All three proteins all contain N-terminal head domains, central rod domains and C-terminal tail domains. The N-terminal head domains are composed of an extreme N-terminal region which is predicted to be unstructured and the plakin domain, which consists of five spectrin repeats (SRs) in periplakin and envoplakin and six SRs in desmoplakin. All three proteins have a Src homology 3 (SH3) domain embedded within a SR5 loop. The numbering and organisation of the SRs are as suggested previously [21]. The C-terminal tail of envoplakin contains a linker region (L) and one plakin repeat domain (PRD) designated C. The periplakin tail consists of a linker alone whereas the desmoplakin tail consists of three homologous PRDs, which are denoted A, B and C respectively, and a linker between PRD-B and PRD-C. The plakin domains (comprised of the residues indicated) of all three proteins were purified and used in all subsequent experiments. (b) Sedimentation velocity profiles of plakin domains of envoplakin (residues 144–891) and periplakin (residues 131–871) reveal monomeric states with molecular masses of 84 kDa in both cases. (c) CD spectra of these plakin domain constructs of envoplakin and periplakin show double-negative minima at 208 and 222 nm, indicating largely α-helical folds.

Envoplakin, periplakin and desmoplakin share a common architecture with a N-terminal head region, a central coiled coil rod domain and a C-terminal tail domain (Fig. 1). The head region of all three proteins is dominated by the plakin domain, which is shared by six mammalian plakin relatives, and in envoplakin, periplakin and desmoplakin consists of a number of spectrin repeat (SR) modules and a Src homology 3 (SH3) domain. An N-terminal extremity which is predicted to be unstructured mediates protein interactions: in periplakin it interacts with plectin [18] and kazrin [19], whereas in desmoplakin it associates with plakoglobin [20]. Desmoplakin’s plakin domain may also be important for protein interactions as a direct interaction between it and plakophilin 1 has been shown *in vitro* [21]. The central coiled coil rod domain mediates heterodimerisation of envoplakin and periplakin [22] and homodimerisation of desmoplakin [23]. The C-terminal tail region contains a variable number of plakin repeat domains that engage intermediate filaments [24], [25]. Thus plakin domains form central connectors that link cell adhesion and cytoskeletal machineries, with their dynamics and structures playing pivotal roles in organising cellular architecture and maintaining the integrity of stressed tissues.

Spectrin repeats have traditionally been viewed as rod-like building blocks that assemble into long extended structures. X-ray diffraction studies of crystallized sets of SRs have revealed exclusively linear structures, including a rigid rod for the first four SRs of the desmoplakin plakin domain [26]. Whether plakin domains act as rigid spacers, or whether they are more flexible like spectrin itself, which has long been considered to be responsible for conferring elasticity on the erythrocyte membrane [27], is not known. We recently showed that the entire plakin domain from desmoplakin forms a non-linear shape in solution, with a long arm comprised of SR3-6 linked with a short arm comprised of SR7-8 [21]. Herein we show that the plakin domains of envoplakin, periplakin and desmoplakin display distinct conformations, and define their distinct ranges of available flexibility. All three proteins are shown to possess a dynamic hinge that allows distinct levels of mobility of the two rigid arms. The plakin domains of envoplakin and periplakin exhibit a restricted ‘helicopter propeller’-type motion whereas desmoplakin’s exhibits a largely unrestricted ‘jack-knife’-type of motion. We propose that these specialised hinges determine how plakin domains contribute to the assembly and stability of desmosomes and the cell cytoskeleton.

## Materials and Methods

### Constructs and Protein Purification

DNA encoding the plakin domains of human envoplakin (residues 144–891) and periplakin (residues 131–871) was cloned in-frame with glutathione S-transferase (GST) in the expression vector pGEX-6P-1 (GE Healthcare). DNA encoding the plakin domain of human desmoplakin (residues 180–1022) was cloned as described [21]. All three plakin DNAs were tagged at the 3′ end with DNA encoding amino acids SGHHHHHH. Envoplakin and periplakin plakin constructs were expressed in *E.coli* strain BL21 (DE3) and purified as described for human desmoplakin [21]. Briefly, fusion proteins were purified by glutathione affinity chromatography and cleaved with PreScission protease. His_6_ tagged plakin domains were then bound to Ni sepharose, washed and eluted with imidazole.

### Analytical Ultracentrifugation

A Beckman XL-1 analytical ultracentrifuge with an eight-cell 50Ti rotor was used for sedimentation velocity analysis of purified proteins. Proteins in 100 mM NaCl and 20 mM sodium phosphate (pH7.4) buffer were loaded into double-sector cells at three concentrations between 0.05 and 4 mg/ml (0.5–35 µM). Samples were centrifuged at 25,000 and 40,000 rpm for 20 h at 20°C and detected at 280 nm. Sedimentation coefficients and molecular masses were determined using the continuous c(s) analysis method and SEDFIT software [28].

### Far-UV Circular Dichroism Spectroscopy

Proteins were dialysed into 20 mM sodium phosphate (pH7.2) buffer and CD spectra were measured at room temperature with a Jasco J-810 spectropolarimeter using a 10 mm path length cuvette. The protein concentrations were 0.1 mg/ml (1–4 µM) and the scanned wavelength range 190–260 nm. Spectra were analysed by DICHROWEB [29] using the CDSSTR algorithm and protein database set 4.

### Small-angle X-ray Scattering

SAXS data were collected at the X33 beamline at the EMBL Hamburg outstation as described [21]. Scattering patterns were collected at room temperature at concentrations between 0.5 and 5.5 mg/ml in 100 mM NaCl and 20 mM sodium phosphate (pH7.2). Background scattering caused by buffer alone was automatically subtracted from the protein scattering profiles. The data were processed using the programme package PRIMUS [30]. Radii of gyration (*R*_g_) and maximum particle sizes (*D*_max_) were determined using GNOM [31].

**Figure 2:**
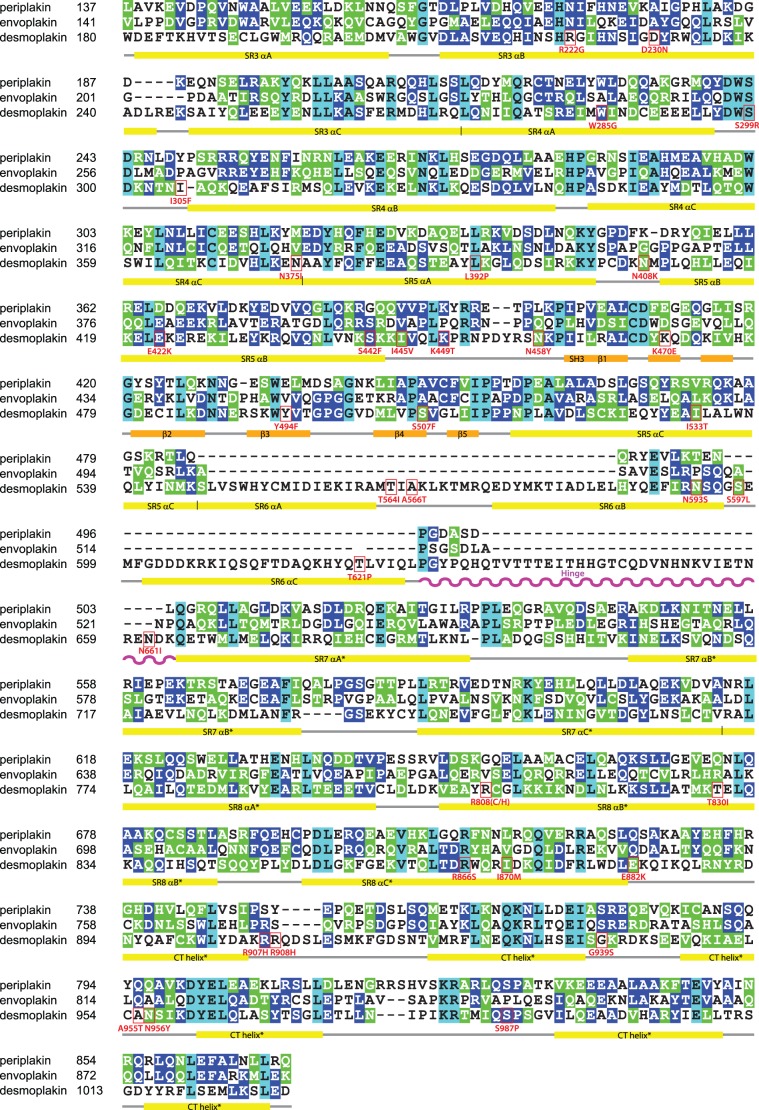
Structure-based sequence alignment of the plakin domains of human periplakin, envoplakin and desmoplakin. Secondary structure elements were obtained from the SR3-6 crystal structure [26]. For the SR7, SR8 and CT regions an asterisk indicates the predicted structural elements based on PSIPRED analysis [41]. Identical, similar and absolutely conserved residues are highlighted in blue, green and cyan, respectively. The α helices are indicated below the spectrin repeats by yellow coloured bars, and the β strands of the SH3 domain with orange bars. The hinge is indicated as a wavy purple line.

In order to gauge the flexibility of the envoplakin, periplakin and desmoplakin plakin domains from their SAXS scattering profiles, three types of models were calculated. Firstly, rigid ‘linear extended SR3-CT’ models were created using the I-TASSER server [32]. The template used for the long arms comprising SR3 to SR5 or SR6 of each plakin domain was the crystal structure of the desmoplakin plakin domain (PDB 3R6N). The template for the short SR78-CT arms of each plakin domain was derived from the crystal structure of repeats 15, 16 and 17 of chicken brain alpha spectrin (PDB 1U4Q). Theoretical SAXS scattering curves were created from the linear extended models and fitted to the experimental scattering data using CRYSOL [33]. Secondly, rigid ‘linear SR3-8′ models were constructed in which all the spectrin repeats were aligned into a linear set. Finally, flexible ‘hinged SR3-8′ models were created where SR3-5/6 and SR78 were rigid entities but separated by a flexible hinge between the two. In the ‘hinged ‘SR3-8′ models the flexible hinge was defined as residues P514-P522 for envoplakin, residues P496-L503 for periplakin and residues P627-K663 for desmoplakin based on the multiple sequence alignment (Fig. 2 and Fig. S1). Flexible CT regions were incorporated into both the ‘linear SR3-8′ and ‘hinged SR3-8′ models [21]. Since both the ‘linear SR3-8′ and ‘hinged SR3-8′ models were deemed to have flexible regions a starting pool of 10,000 randomly generated conformers (the ‘random pool’) was generated using the programme RANCH [34]. The programme GAJOE [34] was then used to create a subset of 50 conformers (the ‘selected ensemble’) that best represented the SAXS scattering curve. The best fit of the selected ensembles to the experimental SAXS scattering data in the form of a curve and a mathematical value (χ^2^) was then calculated by GAJOE. GAJOE was used to calculate *R*_g_ and *D*_max_ values for the random pools and selected ensembles, and distribution graphs comparing the distances between selected plakin domain amino acids in the random pools and selected ensembles were calculated.

## Results and Discussion

### The Plakin Domains of Envoplakin and Periplakin form Intact Helical Monomers

**Figure 3:**
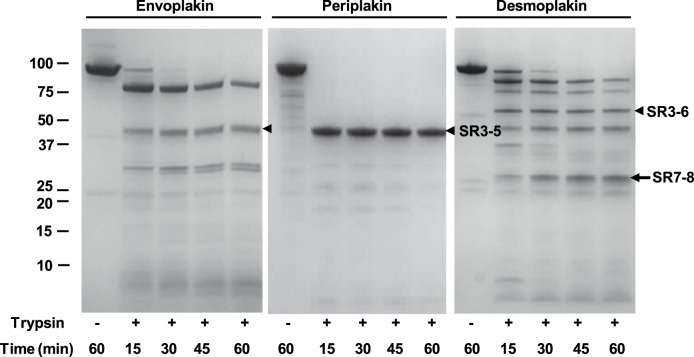
Limited proteolysis of the plakin domains of envoplakin, periplakin and desmoplakin. Proteins were purified and subjected to limited proteolytic digestion with trypsin (0.01 mg/ml) at 37°C. Digestion of envoplakin and periplakin yielded major bands of 42 kDa, beginning with vector-derived GPLGS and native EVDP sequences, respectively, i.e. immediately N-terminal to SR3, and finishing within hinge or SR7 sequences. These bands closely match the expected sizes of the SR3-5 domains, ie. 40.4 and 41.7, respectively, based on the alignment which infers their lack of SR6 modules. Similar cleavage patterns are found with endopeptidase GluC, and are consistent with mass spectrometry results with the protease resistant periplakin SR3-5 fragment (data not shown). Tryptic cleavage of desmoplakin occurred in the flexible hinge at Lys653 and yielded a larger 54 kDa fragment corresponding to SR3-6. This agrees with previous desmoplakin digestion experiments showing that trypsin and chymotrypsin cleave its plakin domain N-terminal to SR3’s αA helix at Lys167 and Tyr172, leaving a 55 kDa proteolytically resistant fragment that encompasses its SR3-SR6 sequence and is resolvable by X-ray crystallography [26]. Digestion of desmoplakin also yielded a 27 kDa SR7-8 protease resistant fragment, beginning with the sequence VIENTR. This fragment could also be expressed as a soluble domain that yielded dispersed NMR spectra indicative of a helical fold [21]. Comparably sized but less abundant fragments were obtained upon digestion of envoplakin and periplakin.

Constructs encompassing the entire plakin domains of human envoplakin, periplakin and desmoplakin (Fig. 1a) were designed based on structure-based alignment of 45 related sequences (Fig. S1). While most spectrin repeats and single SH3 domain are highly conserved (Fig. 2), the sequence alignments revealed that SR6 is absent in all envoplakin and periplakin paralogues, and that the hinge connecting to SR7 is particularly divergent. The three proteins were expressed in *E. coli* with N-terminal glutathione S-transferase (GST) and C-terminal 6xHis tags, purified by glutathione chromatography, cleaved to remove the GST tag and further purified by Ni affinity chromatography (Fig. 3). Envoplakin’s and periplakin’s plakin domains exhibited exclusively monomeric states by analytical ultracentrifugation (Fig. 1b), with sedimentation velocities corresponding to molecular masses of 84 kDa in both cases. This compares favourably with their theoretical molecular masses of 84.0 and 85.1 kDa. Thus all three plakin domains examined, including that of desmoplakin [21], are exclusively monomeric. The conserved monomeric nature of plakin domains, which is in marked contrast to the proposed dimeric nature of adjacent rod domains [35], could endow these terminal domains with relatively unencumbered mobility when the desmosome is assembled or stressed.

The plakin domain structures of envoplakin and periplakin were characterized by far-UV circular dichroism (CD) spectroscopy (Fig. 1c). The expected α-helical folds were evident from the double-negative minima at 208 and 222 nm. A helical content of 67 and 62% was estimated for envoplakin and periplakin, respectively, and β-sheet content of 4 and 9% (Table 1). This matches with desmoplakin’s plakin domain structure [8], and suggests that all three plakin domains share the same overall structure, which is composed of α helical spectrin folds and a single SH3 domain with the expected mixed α/β fold. Thus, whether there are structural explanations for their respective functions remains unclear, with dynamics potentially playing a distinguishing role.

**Table 1:** Secondary structure content of the envoplakin and periplakin plakin domains.

Protein	α-helix	β-sheet	Turn	Disorder
Envoplakin	0.67	0.09	0.10	0.14
Periplakin	0.62	0.04	0.11	0.23
Desmoplakin	0.67	0.08	0.12	0.13

The secondary structure content of each protein was estimated from CD spectra using the DICHROWEB server and is shown along with the previously published results for desmoplakin [21] for comparison.

### Mapping a Flexible Linker

In order to identify the potential regions of flexibility in plakin domains, the sequences of 45 homologues from across vertebrate evolutionary space were aligned based on conservation of structural elements (Fig. S1). This yielded several unexpected results. Firstly, SR6 is missing in all envoplakin and periplakin homologues, which have only a single short predicted helix instead. This infers that there are two major subtypes of plakin domain structures which possess either 5 or 6 canonical SRs, with SR6 being most variable in its presence and sequence. Second, there is a long heterogeneous polar sequence (37 residues in human desmoplakin) that is found after the SR6 repeat of all desmoplakin orthologs, but which is replaced with a very short polar linker (6–8 residues) in all other plakin proteins including envoplakin, periplakin, BPAG-1, MACF1 and plectin (Fig. 2 and Fig. S1). Limited proteolysis was performed on human envoplakin, periplakin and desmoplakin plakin domain proteins to investigate whether this element was exposed. The experiments consistently yielded fragments corresponding to SR3-5/6, and show that a protease sensitive flexible linker is present between the SR5/6 and SR7 structures (Fig. 3).

### Identification of the Flexible Hinges of Envoplakin, Periplakin and Desmoplakin

**Figure 4:**
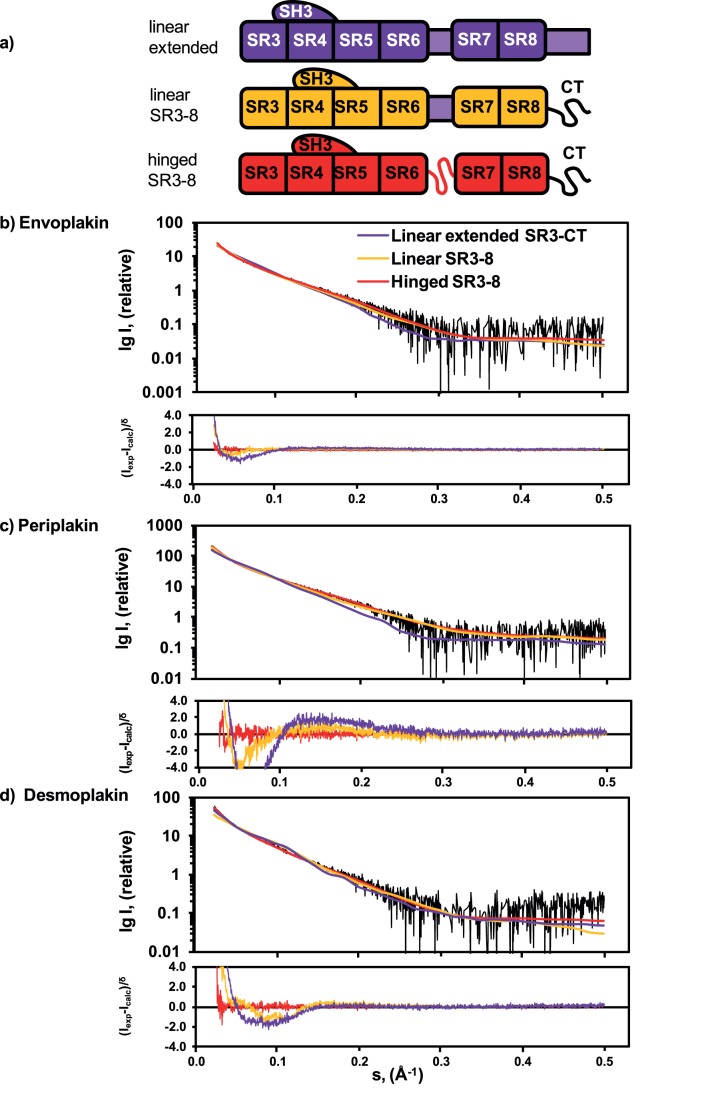
Plakin domains are flexible in solution with a linker between SR6 and SR7. (a) Diagram showing the linear extended SR3-CT model (with no flexible regions and a folded CT region), the linear SR3-8 model (with a rigid α-helical linker between SR5/6 and SR7 and flexible CT region) and the hinged SR3-8 model (with a flexible hinge between SR5/6 and SR7 and a flexible CT region). Rigid elements are shown as boxes and flexible elements as wavy lines. Experimental SAXS scattering data for (b) envoplakin, (c) periplakin and (d) desmoplakin. CRYSOL fits to the experimental scattering data for the linear extended SR3-CT models are shown in purple. GAJOE fits of the selected ensemble to the experimental scattering data for the linear SR3-8 models are shown in yellow. GAJOE fits for the hinged SR3-8 models are shown in red. χ^2^ values reported in Table 2. For each plakin domain a residual plot indicates the level of corroboration of each model to the SAXS scattering data.

Due to their potential importance in plakin protein function we investigated the degree of flexibility in the linkers of plakin domain structures. Initially Kratky plots were created to investigate potential flexibility in each plakin domain. Each plakin domain revealed a peak followed by a slight downward trend indicative of a predominantly structured protein with some flexible elements (Fig. S2). To investigate this further, three different models of all three proteins with different degrees of flexibility were tested using the SAXS data. The ‘linear extended SR3-CT’ model (Fig. 4a) included the canonical SRs as a linear series as well as an additional spectrin repeat fold following SR8 based on earlier inferences that it was SR-like [36]. In contrast, the ‘linear SR3-8′ model includes this C-terminal region (CT) as disordered, consistent with earlier analysis of demoplakin [21]. Both models were kept rigid with an α-helical linker maintaining a linear connection between SR5/6 and SR7. Finally the ‘hinged SR3-8′ model included a flexible linker preceding SR7, as would be consistent with the proteolytic sensitivity of this heterogeneous polar sequence.

Linear extended SR3-CT models gave an unfavourable fit for each of the plakin domains, consistent with previous results on desmoplakin (Fig. 4b,c,d) [21]. This was corroborated by the higher χ^2^ values reported for each fit (Table 2). This implies that a linear extended model was incompatible with the plakin domains’ behaviour in solution. The remaining two models were tested by generating 10,000 random conformations of each protein and applying the Ensemble Optimization Method (EOM) [34]. An ensemble of the 50 conformations that best fit the SAXS data were selected using the Genetic Algorithm Judging Optimization of Ensembles (GAJOE) program. The flexibly ‘hinged SR3-8′ models of all three plakin domain proteins consistently yielded ensembles that fit the experimental data better than the rigid ‘linear SR3-8′ models, especially at lower angles where the overall shape of the protein has its most prominent effect on shape determination (Fig. 4b,c,d). This can be seen more clearly by the residuals plots where the hinged SR3-8 model fits the data better than either the linear extended or linear SR3-8 models. Furthermore hinged SR3-8 models yielded significantly lower χ^2^ values, indicating a better fit and closer representation of SAXS data and actual shape of the protein in solution (Table 2). Thus we infer that all three plakin domains contain flexible hinges and are not held in rigid linear conformations.

**Table 2:** *R*_g_ and *D*_max_ values for each plakin domain.

	GNOM		CRYSOL	EOM			
	*R*_g_ (Å)	*D*_max_ (Å)	χ^2^ (linear extended)	χ^2^ (linear SR3-8)	χ^2^ (hinged SR3-8)	*R*_g_ (Å) (Pool)	*R*_g_ (Å) (Selected)
Envoplakin	75.1	240.0	3.4	1.8	1.2	71.0	63.0
Periplakin	63.4	225.0	5.3	2.1	0.9	75.6	64.0
Desmoplakin	75.1	250.0	3.1	1.9	0.9	80.7	70.3

*R*_g_ and *D*_max_ values were calculated by GNOM from the SAXS scattering data. EOM averaged Rg was calculated for the random pools and selected ensembles as the average of Rg*Rg Rg = √ ([Rg*Rg]). χ^2^ values were calculated using either CRYSOL (for the linear extended model) or EOM (for the linear and hinged SR3-8 models). The hinged SR3-8 model consistently has the lowest χ^2^ value so is more representative of the SAXS scattering data.

### Elucidation of the Unique Conformational Ensembles of Envoplakin, Periplakin and Desmoplakin

**Figure 5:**
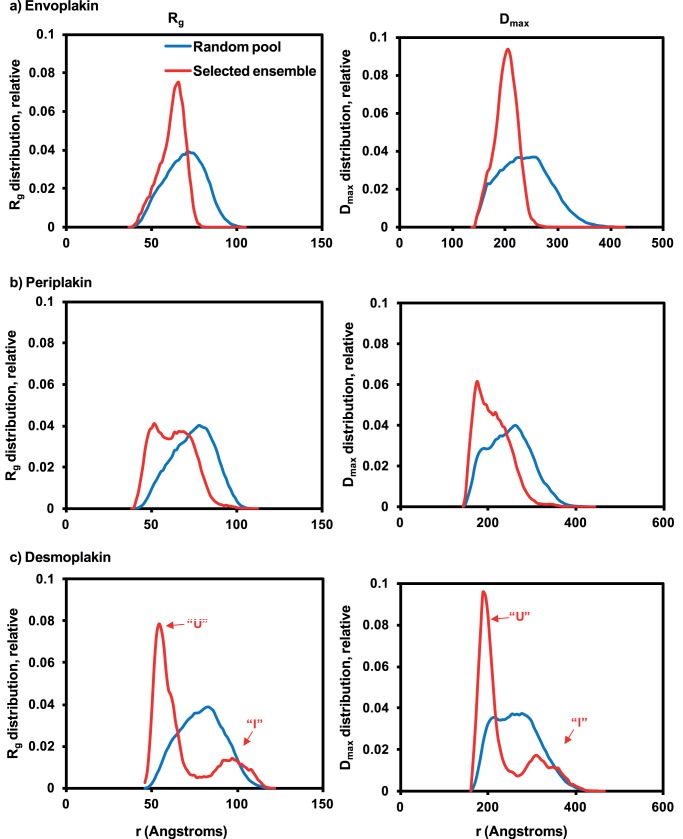
The radii of gyration and maximum particle diameters of plakin domain ensembles. *R*_g_ and *D*_max_ values for the pools of 10,000 random structures generated by EOM (blue lines) and ensembles of 50 structures selected by GAJOE (red lines) for (a) envoplakin, (b) periplakin and (c) desmoplakin.

In order to define the nature of the hinge articulation in the three plakin domains, GAJOE was used to calculate the *R*_g_ and *D*_max_ values for each flexible model’s random pools and selected ensembles (Fig. 5). The average *R*_g_ values estimated from the selected ensembles were similar (periplakin) or lower (envoplakin and desmoplakin) than those obtained by analysis by GNOM (Table 2). The average *R*_g_ values given by EOM are, however, more representative of the data given the plakin domains’ flexible nature [37]. The random pools of all three proteins exhibited Gaussian distributions in terms of both the *R*_g_ and *D*_max_ profiles, indicating similar ranges of potential mobility about the respective hinges. However, the best ensemble of envoplakin structures selected by GAJOE fitting displayed a much narrower *R*_g_ distribution with a 8 Å reduction on average, indicating restricted range of mobility. In contrast, desmoplakin’s selected ensemble revealed a pair of peaks in both the *R*_g_ and *D*_max_ profiles, suggesting two dominant orientations at either extreme of the articulation range. Finally, periplakin exhibited an intermediate profile by both measures, inferring that its hinge allowed mobility that was freer than envoplakin but tighter than desmoplakin.

Two major conformers were evident in the selected ensemble of desmoplakin structures based on the pair of peaks for both the *R*_g_ and *D*_max_ profiles (Fig. 5). Of these, the first peaks showed a large shift to the left when compared to the random pool peaks, indicating that the desmoplakin plakin domain has a marked propensity to adopt a ‘U’ shaped conformation. The second peaks were shifted to the right when compared to the random pool peaks, suggesting that the desmoplakin plakin domain can also adopt an extended linear ‘I’ conformation. The populations of first ‘U’ peaks from both the *R*_g_ and *D*_max_ profiles were more than three times greater than that of the second ‘I’ peaks, indicating that the compact conformation predominates over the extended conformation (Fig. 5). Overall, this indicates that the hinge regions of all three plakin domains are flexible, and that envoplakin and periplakin tend to adopt ‘L’ shaped conformations in solution whereas that of desmoplakin has a marked propensity to adopt a ‘U’ shaped orientation.

**Figure 6:**
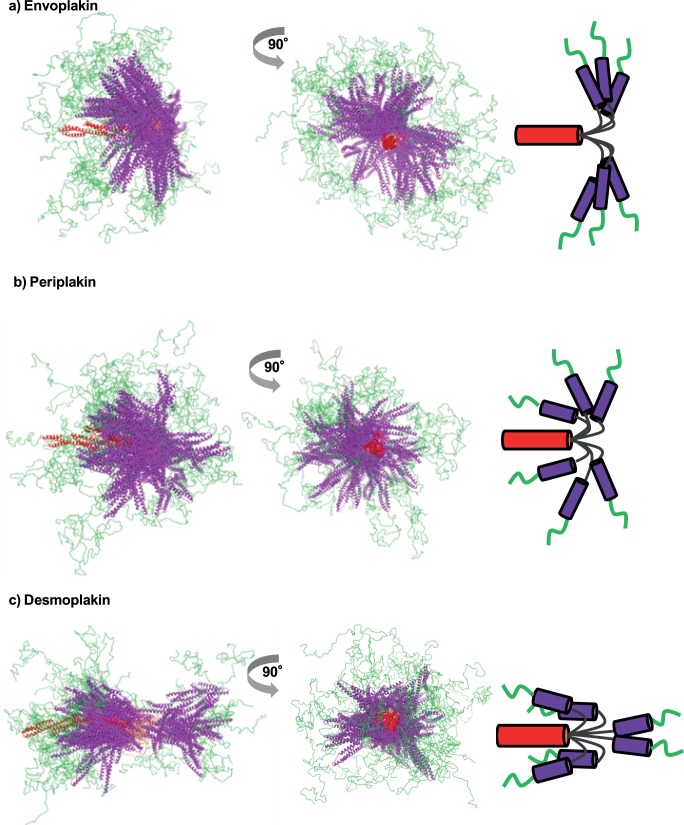
Molecular representations of the three plakin domains based on EOM selected conformers. The selected ensembles of only the 50 best structures which were selected by GAJOE are shown in two orthogonal views for (a) envoplakin, (b) periplakin and (c) desmoplakin. Also indicated are cartoon representations of the predominant conformers. The SR3-6 rods (red) were aligned and the SR7-8 rods (purple) and the CT region (green) allowed to rotate freely about the hinge (gold). C-terminal residues of SR8 are coloured red. Also indicated are cartoon representations of the predominant conformers.

Two predominant ranges of motion are evident, and can be described as helicoptering and jack-knifing. Representations of the selected ensembles of the 50 plakin domain conformations that best fit the scattering data are shown in Fig. 6. In order to illustrate the flexibility of the hinge, the long SR3-5/6 plakin arms were held in place with the SR7-8-CT arms left free to rotate about the hinge. Envoplakin and periplakin exhibit ‘helicopter propeller’-like distribution profiles with SR78 showing a relatively tight right angle distribution of orientations about the hinge. By contrast desmoplakin appears like a jack-knife, with most of the conformations adopting a ‘U’ shape in which the SR78 region becomes anti-parallel to SR3-5/6 arm, with the remainder being in a linear ‘I’ orientation. For each of the three plakin domains the distance between the first amino acid of SR3 and the last amino acid of SR8 was calculated for the random pools and selected ensembles generated for the hinged SR3-8 model (Fig. S3). For desmoplakin the amino acid distribution curve of the selected ensemble showed a similar profile to those observed for *R*_g_ and *D*_max_ (Fig. 5), consistent with a largely dual existence of either contracted or extended conformers with either very short or long distances between first and last residues. The distribution curves for the envoplakin and periplakin selected ensembles are both shifted to the left when compared to the distribution curves of their random pools confirming that a ‘L’ shape, not a linear ‘I’ conformation, is the predominant form of these proteins.

Overall, our data reveal that plakin domains exhibit specialized flexibility, with desmoplakin having a particularly wide range of motion around its long hinge. In contrast the range of motion about the shorter hinges of periplakin and especially envoplakin is restricted. A distinct hinge sequence is found in the plakin domains of BPAG-1, MACF and plectin (Fig. S1), being short but much more highly charged than those of envoplakin and periplakin, suggesting yet another mode of articulation. As such the hinge represents a distinguishing feature of different plakin domains and may help to define their respective functions.

Recent X-ray crystallographic data show that the first four SRs of desmoplakin form an elongated, rigid structure [26]. Although no such high resolution data exists for SR7 and S8, they are seen to form a linear rod by SAXS [21]. Furthermore, it may be that some limited flexibility within the short arm is conferred by the short region that joins SR7 and SR8 [38]. We suggest that specialised hinges within plakin domains allow focussed flexibility between sets of helically-connected SR rods which are themselves relatively inflexible. A similar situation is thought to exist in dystrophin, which contains multiple proline-rich hinges between the stiff rods formed by subsets of its 24 SR’s [39]. These hinges could act like accordion-like springs, allowing extension and compression when the molecule is subjected to mechanical force, thus limiting SR unfolding [39]. The long desmoplakin hinge could act as a particularly adaptable “universal joint”, affording greater protection to the integrity of the desmosomal machinary in mechanically stressed tissues. Multiple hinges could work in parallel in the homodimers formed by desmoplakin through its adjacent rod domain [23]. The shorter hinges of envoplakin and periplakin would facilitate more controlled mobility, and would be in register in the parallel homo- and hetero- dimers mediated through their paired rod domains within the desmosome [22].

The flexibility of the plakin domain could determine how its protein partners in cell junctions become juxtaposed. Desmoplakin’s N- and C- termini link to plakoglobin and plakophilins, and intermediate filaments, respectively [24], [40], with the ‘I’ and ‘U’ plakin domain conformers being able to draw these partners into distal or proximal positions within the desmosome. Similarly, the inherent flexibility of the plakin domains of envoplakin and periplakin may be important for assembly of these proteins into complexes on the membrane during the formation of the cornified envelope [6]. Overall, we suggest that plakin domains are functionally defined by their unique flexibilities, which allows dynamic yet stable connections between the plasma membrane and the cell cytoskeleton. Our results provide the first experimental evidence that shows that articulating hinges are defining features of plakin domains, and provide a molecular explanation as to how they could contribute to junction assembly, and elasticity and stability in tissues exposed to mechanical stress.

## Supporting Information

Figure S1 Alignment of the SR4, SR5, SH3, SR6 and αA helix of SR7 sequences of periplakin, envoplakin, desmoplakin, BPAG-1 MACF1 and plectin. Starting positions of the SR helices and SH3 domain are indicated. Each protein name is followed by the first letters of the following genus and species: Ailuropoda melanoleuca, Anolis carolinensis, Bos Taurus, Canis familiaris, Danio rerio, Equus caballus, Felis catus, Gallus gallus, Homo sapiens, Monodelphis domestica, Mus musculus, Oreochromis niloticus, Sus scrofa, Xenopus tropicalis. Residues that are absolutely conserved across all sequences, identical amongst most sequences, or similar across most sequences are highlighted in cyan, blue and green, respectively. The sequence alignment was performed using the ClustalW program [42] at the SDSC Molecular Biology Workbench, and colouring was performed using Boxshade version 3.3.1, which was developed by Kay Hofmann and Michael D. Baron, based on the ClusterW 1.60 similarities.
(TIF)

Figure S2 Kratky plots of the envoplakin, periplakin and desmoplakin plakin domains. The Kratky plots (I(s)/I/0*s^2^) of the plakin domains of (a) envoplakin, (b) periplakin and (c) desmoplakin were used to investigate the potential flexibility. A subtle peak is observed at a value of 1 followed by a slight downward trend indicative of a predominantly folded protein with flexible elements for each plakin domain.
(EPS)

Figure S3 Distance between the first residue of SR3 and the last residue of SR8 for all three plakin domains. Distance distributions were calculated for the random pools (blue) and selected ensembles (red) for (a) envoplakin (P144-V746); (b) periplakin (E131-L725); (c) desmoplakin (W180-L881).
(EPS)
