# Supplementary figures and images for: Hinged Plakin Domains Provide Specialized Degrees of Articulation in Envoplakin, Periplakin and Desmoplakin
Source: PLoS One. 2013 Jul 29;8(7):e69767. doi: 10.1371/journal.pone.0069767 (PMC3726778; doi:10.1371/journal.pone.0069767)

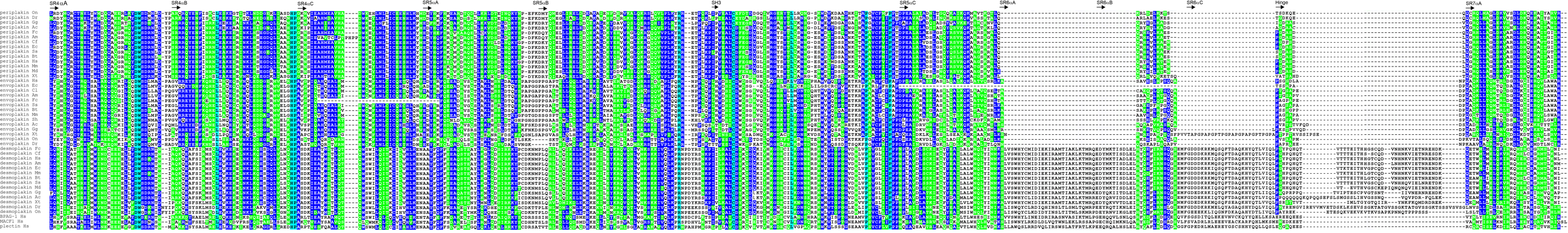

Supplement: Figure S1 — Starting positions of the SR helices and SH3 domain are indicated. Each protein name is followed by the first letters of the following genus and species: Ailuropoda melanoleuca, Anolis carolinensis, Bos Taurus, Canis familiaris, Danio rerio, Equus caballus, Felis catus, Gallus gallus, Homo sapiens, Monodelphis domestica, Mus musculus, Oreochromis niloticus, Sus scrofa, Xenopus tropicalis. Residues that are absolutely conserved across all sequences, identical amongst most sequences, or similar across most sequences are highlighted in cyan, blue and green, respectively. The sequence alignment was performed using the ClustalW program [42] at the SDSC Molecular Biology Workbench, and colouring was performed using Boxshade version 3.3.1, which was developed by Kay Hofmann and Michael D. Baron, based on the ClusterW 1.60 similarities. (TIF) [file pone.0069767.s001.tif]
